# Body Mass Index and Related Risk Factor of Sinusitis Among Adults in Saudi Arabia: A Cross-Sectional Study

**DOI:** 10.7759/cureus.40454

**Published:** 2023-06-15

**Authors:** Abdullah D Alotaibi, Mubashir Zafar, Bashayr N Alsuwayt, Rana N Raghib, Abeer H Elhaj

**Affiliations:** 1 Otolaryngology - Head and Neck Surgery, University of Hail College of Medicine, Hail, SAU; 2 Community Medicine, University of Hail College of Medicine, Hail, SAU; 3 Medicine and Surgery, University of Hail College of Medicine, Hail, SAU; 4 Family and Community Medicine, University of Hail College of Medicine, Hail, SAU

**Keywords:** rhinitis, diabetes, smoking, crs, chronic rhinosinusitis, sinusitis, bmi, saudi arabia

## Abstract

Background: Chronic rhinosinusitis (CRS) is a widespread condition worldwide that is leading to a significant burden on society in terms of healthcare consumption and productivity loss. Multiple risk factors have been implicated in the pathogenesis of CRS, such as nasal allergies, bronchial asthma, smoking, nasal polyps, and immune system-related diseases. The present study aimed to assess the prevalence of CRS, the most common risk factors, and the association between diabetes, body mass index (BMI), and CRS in Saudi Arabia.

Methods: A cross-sectional study was conducted through random sampling that included 3602 participants from different regions of Saudi Arabia from November 2022 to January 2023. Electronic questionnaires were used for data collection.

Results: A total of 3602 individuals responded to our questionnaires; 948 (26.3%) were diagnosed by physicians as having chronic rhinosinusitis, and 75.1% were females. The majority (41.5%) were between the ages of 18 and 28 years. We found that smoking was significantly associated with sinusitis (OR 2.01, 95% CI 1.23-5.69) (p value 0.003) and that obesity was also significantly associated with sinusitis, 17.0% of persons with sinusitis were obese class I (BMI 30-35 kg/m^2^); 7.8% were obese class II (BMI>35 kg/m^2^); and 29.4% were overweight, whereas 45.8% were of normal weight. All percentages differ significantly from persons with normal weight (p value = 0.000). The most common risk factors for sinusitis were nasal allergies (44.4%), stuffy nose (22.8%), and deviation of the nasal septum (19.2%). All percentages differ significantly from persons without comorbidity (p value = 0.000).

Conclusion: The present study showed a slight increase in the prevalence of CRS in Saudi Arabia, which is attributable to increased exposure to allergens. The most common risk factors were nasal allergies, nasal blockage, deviation of the nasal septum, and asthma. There was a significant correlation between CRS and BMI in the form of increased prevalence in overweight and obese compared to normal-weight individuals.

## Introduction

Worldwide, rhinosinusitis (RS) is a common illness that places a heavy burden on society in terms of healthcare spending and lost productivity [1]. Pathologically, rhinosinusitis is described as a persistent, transitory inflammation of the sinus mucosa. According to its duration, RS is split into three categories: acute rhinosinusitis (ARS) defined as lasting less than 12 weeks; recurrent acute rhinosinusitis (RARS) defined as lasting four or more episodes in a year [2-3]; and CRS lasting longer than 12 weeks. The last one is further divided into CRS without nasal polyps (CRSsNP) and CRS with nasal polyps (CRSwNP) based on clinical phenotype [4]. Even with treatment, the sinuses may remain swollen and inflamed for three months or longer. Chronic sinusitis is characterized by swelling of the inner lining of the sinuses, which causes discomfort and swelling around the eyes and head as well as breathing difficulties. This widespread condition prevents mucus from draining and results in stuffy noses. Chronic sinusitis is a disorder that can be brought on by an infection, nasal polyps, or swelling of the sinus lining. Other signs and symptoms are referred to as chronic rhinosinusitis.

Sinusitis may impact both children and adults. In addition to bringing on colds, allergens can raise the risk of developing chronic sinusitis. Various factors such as viruses, fungi, or bacteria can bring on chronic sinusitis, which can last for months or even years [5]. Aspirin intolerance, nasal polyposis, and chronic sinusitis are all related [6,7]. Nearly 20% of chronic sinusitis patients also have nasal polyposis [8].

The estimated prevalence of CRS is 3.0-6.4% in the general population [3]. The prevalence of CRS, based on self-reported symptoms, has been estimated at (10.9%) in a European multicenter study [9], 8.2% in a cross-sectional survey in China [10], and 5.5% in Brazil [11]. The prevalence of CRS in Saudi Arabia is 25.3% [12]. Risk factors increase the chances of developing a sinus infection. Also, anyone can get infected with or without these factors, so we note that the higher the risk, the greater the chance that a person will develop the disease.

Certain medical disorders increase a person's risk of developing sinusitis. These include some chronic conditions such as diabetes mellitus (DM), obesity, cystic fibrosis, granulomatosis with polyangiitis, and granulomata (the women were noted to have a higher risk of chronic sinusitis than men), the recent use of decongestant sprays for long-term colds, nasal obstruction caused by polyps and a deviated septum. In addition, other risks are present, including facial bone anomalies, swelling adenoids, cleft palate, and tumors. Furthermore, exposure to environmental factors that increase the incidence of CRS includes smoking, passive smoking, air pollution, flying, and scuba diving [13]. Moreover, genetic diseases such as cystic fibrosis, primary immunodeficiencies, and primary ciliary dyskinesia (Kartagener's syndrome) are linked to a high prevalence of CRS [14]. 

We thought additional research is required to independently confirm findings and uncover connections between CRS, diabetes, obesity, and smoking. are used to assess the severity of the disease and to comprehend the effects of the risk factors in the context of CRS. Because few recent studies specifically address diabetes, obesity, or smoking as risk factors for chronic sinusitis, the goal of this study was to look into the prevalence of chronic sinusitis in Saudi Arabia and the age groups, genders, and risk factors that are most affected.

## Materials and methods

Study design and sample

This study was cross-sectional and exploratory in nature to assess the prevalence of sinusitis and its risk factors among the Saudi population. The Research Ethics Committee (REC) at the University of Hail (21/11/2022) approved the study protocol (reference number: H-2022-395 ), and the study was conducted in conformity with the Helsinki Declaration. The questionnaire was created in Google Forms and made available online by public link, shared via several social media platforms (i.e., Twitter, Facebook, WhatsApp, and Instagram). Saudi citizens and residents (>18 years old) were asked to complete the questionnaire. Participants were encouraged to distribute the survey link to their networks using social media or instant messaging services within the same time frame. The survey was voluntary, and all responses were kept confidential. At the start of the questionnaire, the study's goals and rationale for data collection were stated explicitly. Participants’ consent was also recorded online before any responses were provided. The survey was distributed on November 26, 2022. The intended sample size was determined after a month of data collection. The study involved the distribution of 3602 self-administered online questionnaires to Saudi adult participants.

Data collection and analysis

A self-administered questionnaire was designed with multiple-choice format questions. It consisted of eight questions guided by the study objectives. The questionnaire included two sections. The first comprised demographic information about the participants: age, gender, level of education, occupation, and location. The second section included the prevalence of sinusitis (diagnosed), the symptoms the participant experiences that were associated with sinusitis., the calculation of body mass index by height and weight, the diagnosis of patients who had symptoms but had not been diagnosed by a doctor, and the risk factors that increased the incidence of sinusitis. Data were entered and analyzed using the Statistical Package for Social Sciences (SPSS), version 23.0 (IBM Corp., Armonk, NY).

## Results

Table 1 shows the following results. The prevalence of CRS was 26.3% among people diagnosed by physicians in Saudi Arabia, most of whom were female (75.1%). The age of most participants diagnosed with CRS ranged from 18 to 28 years old (41.5%), followed by participants ranging from 40 to 50 years (26.6%), 29-39 years (24.5%), and more than 50 years (7.4%). Many participants were from the northern area of Saudi Arabia, followed by the western, then the middle areas. Concerning level of education, most participants with CRS had a university degree (68.2%). Regarding nationality and employment, most participants in CRS were Saudi and employed. 

**Table 1:** Baseline characteristics of study participants

Characteristics	Answers Frequency n (%)				Total no. n(%)
	Yes (Total number)		No (Total number)		
	n	%	n	%	
Prevalence of Sinusitis	948	26.3	2654	73.7	3602(100)
Gender					
Male	236	24.9	713	26.9	949(26.3)
Female	712	75.1	1941	73.1	2653(73.7)
Age (years)					
18-28	394	41.5	1687	63.6	2081(57.8)
29-39	232	24.5	403	15.2	635(17.6)
40-50	252	26.6	398	15.0	650(18.0)
>50	70	7.4	166	6.2	236(6.6)
Location in Saudi Arabia					
North	333	22.0	1180	44.5	1513(42.0)
South	34	3.6	99	3.8	133(3.7)
Eastern	145	15.3	327	12.3	472(13.1)
Western	253	26.7	658	24.8	911(25.3)
Middle	183	19.3	390	14.7	573(15.9)
Level of Education					
Primary	10	1.1	29	1.1	39(1.1)
Middle	15	1.6	65	2.4	80(2.2)
Secondary	144	15.2	570	21.5	714(19.8)
University	657	69.2	1798	67.7	2455(68.2)
Above University	122	12.9	192	7.3	314(8.7)
Employment Status					
Student	293	30.9	1350	50.9	1643(45.0)
Employee	449	47.4	812	30.6	1261(35.0)
Unemployment	159	16.8	393	14.8	552(15.3)
Retired	43	4.5	85	3.2	128(3.5)
Dredging worker	4	0.4	14	0.5	18(0.49)
Nationality					
Saudi	892	94.1	2516	94.8	3408(94.6)
Non-Saudi	56	5.9	138	5.2	194(5.4)

Table 2 shows the following results. Different risk factors were associated with sinusitis. After adjustment of co-variates in logistic regression, the age groups 18-28 years and 29-39 years were significantly associated with sinusitis (OR 1.60 {95% CI 1.06-2.40} {p value 0.022}), (OR 1.78 {95% CI 1.30-2.43} {p value 0.000}). Smoking (OR 2.01 {95% CI 1.23-5.69} {p value 0.003}); nasal sensitivity (OR 2.29 {95% CI 1.63-3.22} {p value 0.000}); and exposure to plants and dust (OR 1.05 {95%CI 1.01-2.96} {p value 0.023}) were also significantly associated with sinusitis.

**Table 2:** Association between sinusitis and its risk factors among participants (n=3602)

Characteristics	Sinusitis Present Crude OR (95% CI) (p value)	p value	Sinusitis Present Adjusted OR (95% CI) (p-value)	p value
Gender				
Male	1		1	
Female	0.90 (0.761-1.07) (0.237)	0.237	0.81 (0.65-1.01) (0.071)	0.071
Age (years)				
>50	1		1	
18-28	1.80 (1.33-2.43) (0.000)	0.000	1.60 (1.06-2.40) (0.022)	0.022
29-39	0.372 (0.53-1.01) (0.059)	0.059	1.78 (1.30-2.43) (0.000)	
40-50	0.66 (0.48-1.91) (0.052)	0.052	1.00 (0.77-1.32) (0.071)	0.071
Level of Education				
Primary	1		1	
Middle	2.28 (0.88-5.89) (0.089)	0.089	1.19 (0.21-6.48) (0.838)	0.838
Secondary	2.08 (0.948-4.57) (0.068)	0.068	0.65 (0.34-1.25) (0.202)	0.202
University	1.44 (0.66-3.11) (0.354)	0.354	0.94 (0.48-1.83) (0.864)	0.864
Above University	0.84 (0.37-1.87) (0.673)	0.673	0.57 (0.19-1.70) (0.322)	0.322
Employment Status				
Employee	1		1	
Student	2.54 (2.14-3.02) (0.000)	0.000	1.26 (0.95-1.68) (0.105)	0.105
Unemployment	1.37 (1.11-1.70) (0.004)	0.004	1.10 (0.84-1.44) (0.462)	0.462
Retired	1.10 (0.75-1.62) (0.607)	0.607	0.91 (0.55-1.48) (0.70)	0.700
Nationality				
Non-Saudi	1		1	
Saudi	1.14 (0.83-1.57) (0.408)	0.408	0.85 (0.58-1.23) (0.40)	0.40
Smoking				
No	1		1	
Yes	1.12 (1.01-12.52) (0.051)	0.051	2.01 (1.23-5.69) (0.003)	0.003
Co-Morbidity				
None			1	
Deviation of the nasal septum	1.21 (0.78-1.87) (0.390)	0.390	1.06 (0.66-1.70) (0.791)	0.791
Stuffy nose/Nasal Sensitivity	2.77 (2.04-3.76) (0.000)	0.000	2.29 (1.63-3.22) (0.000)	0.000
Asthma	0.35 (0.25-0.49) (0.000)	0.000	0.34 (0.23-0.49) (0.000)	0.000
Diabetes	0.25 (0.17-0.37) (0.000)	0.000	0.24 (0.16-0.37) (0.000)	0.000
Environment				
None	1		1	
Gases & Fumes	0.83 (0.51-1.35) (0.462)	0.462	1.10 (0.71-1.70) (0.665)	0.665
Moist Environment	0.80 (0.52-1.25) (0.339)	0.339	0.833 (0.48-1.43) (0.510)	0.510
Pet breeding	0.66 (0.44-1.00) (0.052)	0.052	0.93 (0.57-1.53) (0.801)	0.801
Frequent exposure to detergents	1.44 (1.02-2.89) (0.045)	0.045	0.692 (0.43-1.09) (0.118)	0.118
Plants & Dust	1.61 (1.09-2.37) (0.016)	0.016	1.05 (1.01-2.96) (0.023)	0.023
BMI (kg/m^2^)				
Normal Weight (<25)	1		1	
Overweight (25-29.9)	1.49 (1.11-1.98) (0.005)	0.005	0.93 (0.66-1.32) (0.708)	0.708
Obese Class I (30-35)	1.12 (0.83-1.51) (0.437)	0.437	1.01 (0.71-1.42) (0.955)	0.955
Obese Class II (>35)	1.03 (0.74-1.14) (0.837)	0.837	1.03 (0.71-1.48) (0.872)	0.872

**Figure 1:**
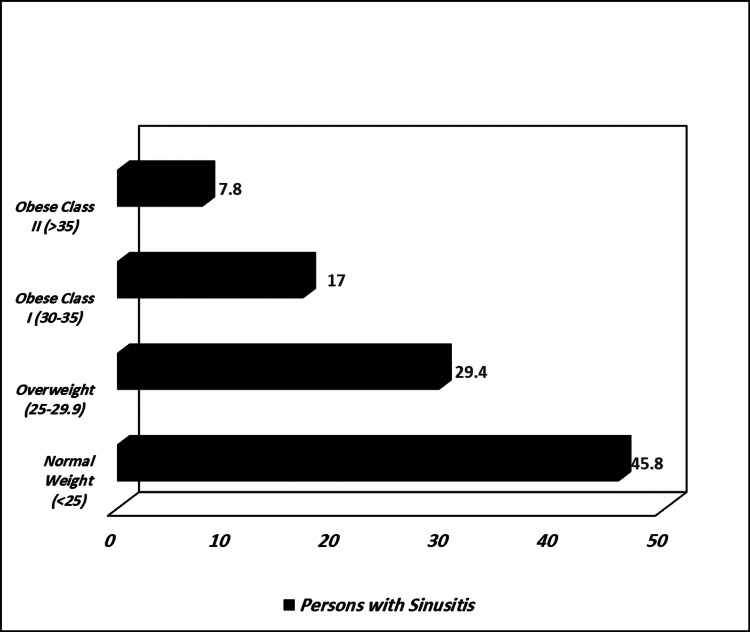
Prevalence of sinusitis among different classifications of BMI (%) BMI: Body Mass Index

Figure 1 shows that 17.0% of people with sinusitis were obese class I (BMI 30-35 kg/m^2^); 7.8% were obese class II (BMI >35 kg/m^2^); 45.8% were overweight; and 29.4% were normal weight. All percentages significantly differ from those of people with normal weight (p value = 0.000).

**Figure 2:**
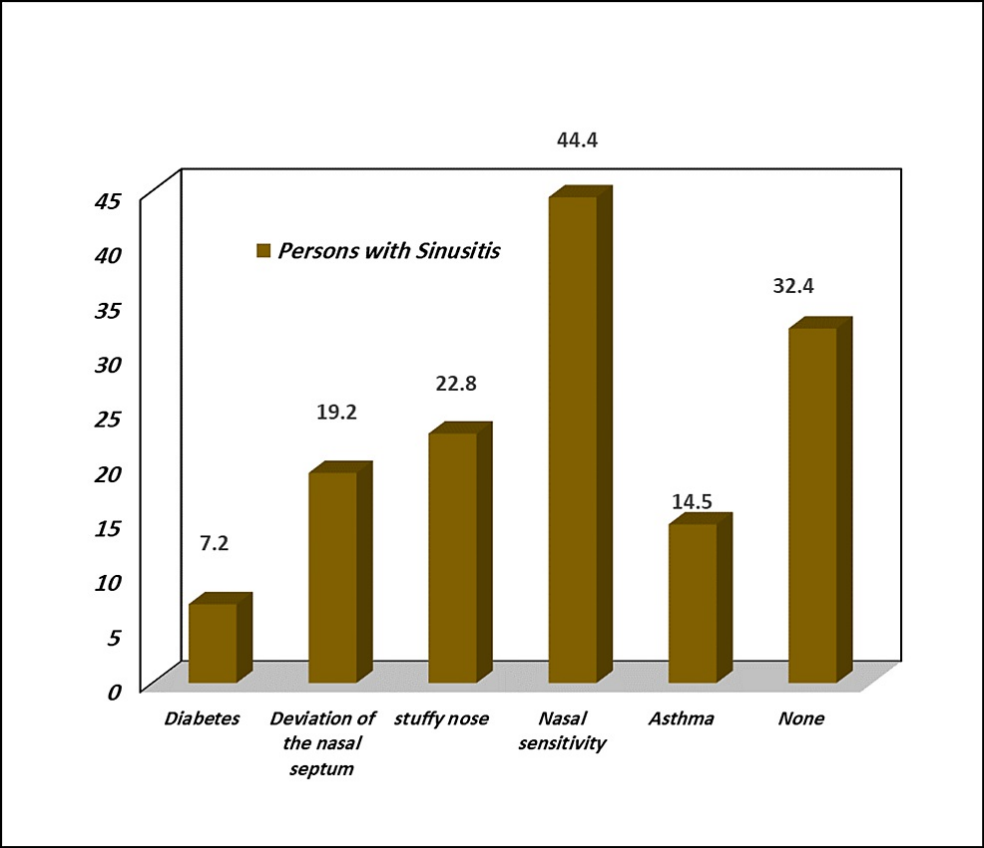
Prevalence of sinusitis with co-morbidity (%)

Also, among people with sinusitis, 44.4% had nasal sensitivity, 22.8% had a stuffy nose, and 19.2% had a deviation of the nasal septum; 14.5% had asthma, and 7.2% had diabetes; all percentages are significantly different from persons without comorbidity, as shown in Figure 2 (p value = 0.000). 

Table 3 shows the following results. According to symptoms of sinusitis experienced by study subjects who were diagnosed with sinusitis (948), the study indicated that about (25.9%) had two or more symptoms for 12 weeks or more. These symptoms were runny nose, secretions from the nose to the throat, stuffy nose, facial pain or pressure, weakness, and loss of smell. Meanwhile, (22.6%) had one symptom, and (51.4%) had no symptoms. Regarding other symptoms, such as headaches, ear pain or pressure, fever, fatigue, bad breath, toothache, and cough with sinusitis, about (27.3%) of participants had two or more symptoms for 12 weeks or more, while (26.4%) had one symptom and (45.6%) had no symptoms.

**Table 3:** The clinical symptoms of sinusitis among participants with and without sinusitis

Question 3		Answers Frequency n (%)		
	Yes (Total number)		Total no. n (%)	
	n	%	n	%
Participants diagnosed with sinusitis	948	26.3	3602	100
Have you had any of the following symptoms for 12 weeks or more: runny nose, secretions from nose to throat, stuffy nose, facial pain or pressure, weakness, or loss of smell?				
Yes, I have had two or more of these symptoms.	520	54.9	933	25.9
Yes, I have had one symptom only.	240	25.3	817	22.6
No, I have not had any symptoms.	188	19.8	1852	51.4
Do you have any of the following symptoms for 12 weeks or more: headache, ear pain or pressure, fever, fatigue, bad breath, toothache, or cough?				
Yes, I have had two or more of these symptoms	465	49.1	984	27.3
Yes, I have had one symptom only	285	30.1	972	26.9
No, I have not had any symptoms.	198	20.9	1646	45.6

## Discussion

In the Saudi population, sinusitis currently affects 26.5% of the population [5]. In 2016, it was 25.3% [5], which was already considered very high. The estimated prevalence of CRS is 3.0-6.4% in the general population [3]. The prevalence of CRS based on self-reported symptoms has been estimated at 10.9% in a European multicenter study [9], 8.2% in a cross-sectional survey in China [10], and 5.5% in Brazil in South America [11]. There is some evidence that CRS is more common in warmer regions than in colder ones, without discounting the impact of weather on this disparity [15]. As shown in a previous study, the prevalence of sinusitis was more common in females than males [9].

The majority of the present study participants with sinusitis were young adults, with ages ranging from 18 to 28 years old (57.8%), followed by those between 29 and 50 years old (35.6%). Those older than 50 years old (6.6%) had a very low prevalence rate. Regarding education, the study found that 19.8% of sinusitis subjects had completed secondary school and 68.2% had graduated from a four-year university. The participants' employment rates were 35.49% and 45.6%, respectively. According to the study's analysis of participant distribution across the government, the majority of participants are from the north of Saudi Arabia, followed by the western and central regions. The incidence of sinusitis was the same, regardless of location.

The present study found that about 25.9% of study participants who were diagnosed with sinusitis (948) had two or more symptoms for 12 weeks or more, including runny nose, secretions from the nose to the throat, stuffy nose, facial pain or pressure, weakness, or loss of smell, while 51.4% were symptom-free and 22.6% experienced just one symptom. Other signs and symptoms include a cough with sinusitis, a headache, ear pain or pressure, exhaustion, foul breath, and toothache. Approximately 27.3% of participants reported two or more symptoms for 12 weeks or longer, compared with 26.4% who had one symptom and 45.6% who had none. A link between smoke exposure and sinusitis was discovered (OR 2.01 {95% CI 1.23-5.69}; {p-value 0.003}). Additionally, smoking has been identified as the environmental component that aggravates sinusitis the most [16].

Overweight and chronic rhinosinusitis are linked, according to previous cross-sectional investigations [17-19]. In our investigation, we discovered a significant relationship between obesity and sinusitis (p value = 0.000), as well as a correlation between greater BMI and paranasal sinus illness [20] compared with people who are of normal weight. We discovered that 54.2% of sinusitis sufferers were overweight. Sinusitis patients made up 17.0% of the obese class I population (BMI 30-35 kg/m^2^) and 7.8% of the obese class II population (BMI >35 kg/m^2^), respectively, whereas 29.4% and 45.8% were overweight or normal weight. In Saudi Arabia, there is a significant prevalence of overweight (BMI >25 kg/m^2^) in both men and women (30.7% vs. 28.4%, respectively), as well as a higher incidence of obesity in women (23.6 vs. 14.2%) [21]. Also, 7.2% of sinusitis patients were diabetic patients, compared with another study, in which 6.09% of sinusitis patients were diabetic [19].

According to a study conducted in Taif City, Saudi Arabia, there was a link between allergic rhinitis and sinusitis. Chronic sinusitis was the most prevalent concomitant condition in people with allergic rhinitis [22]. In our study, allergic rhinitis affected 44.4% of individuals with sinusitis. As a result, in the present study, allergic rhinitis was the most frequent sinusitis risk factor.

Study limitations

Several important limitations need to be considered. First, this was a questionnaire-based study, so it may not have been easily accessible to potential participants from all social classes. Second, some participants may have incorrectly reported their information. Third, the study did not evaluate whether the sinusitis developed before or after increased BMI. In addition, a larger and more widespread study is required to confirm that a high BMI is a risk factor for developing sinusitis. The content evaluated was mainly based in the Kingdom of Saudi Arabia, and our findings may not be as relevant to other countries.

Finally, our study has raised multiple issues that need further investigation. Future research should explore the high association between sinusitis and rhinitis and evaluate the exact pathophysiology behind high BMI as a risk factor for sinusitis. Last, identifying certain associations between environmental risk factors and the development of sinusitis also needs further investigation.

## Conclusions

Our study showed a slight increase in the prevalence of CRS in Saudi Arabia, with females being the most affected. The prevalence of sinusitis was 26.3%, which may be attributed to increased exposure to allergens. The most common risk factors were nasal allergies, nasal blockage, and deviation of the nasal septum, followed by smoke exposure as an environmental risk factor. There is a significant correlation between CRS and BMI in the form of increased prevalence in overweight and obese individuals compared with normal-weight individuals.

## Appendices

**Table 4:** Study questionnaire.

Introduction to the Questionnaire	
We are a research group from the College of Medicine at the University of Hail. We are pleased to invite you to participate in filling out a questionnaire that serves our study, which aims to measure the prevalence of sinusitis and its risk factors in Saudi Arabia in 2022. By filling out the questionnaire, you agree to participate in it.	
Age:	18–28
	29–39
	40–50
	>50
Gender	Female
	Male
Nationality:	Saudi
	Non-Saudi
Accommodation:	Central Region
	Northern region
	Eastern Province
	Western Region
Educational level:	Pre-school
	Middle school
	High school
	University
	Post-graduiate
	Non-educated
Occupation	Student
	Employed
	Craft worker
	Retired
	Housewife
Have you had any of the following symptoms for 12 weeks or more: runny nose, secretions from the nose to the throat, stuffy nose, pain or pressure in the face, or weakness or loss of sense of smell?	Yes, I have two or more symptoms.
	Yes, I only have one symptom.
	No.
Have you had any of the following symptoms for 12 weeks or more: headache, pain or pressure in the ear, fever, fatigue, bad breath, toothache, or cough?	Yes, I have two or more symptoms.
	Yes, I only have one symptom.
	No.
Have you been diagnosed with sinusitis?	Yes.
	No.
are you a smoker?	Yes
	No, but I have been exposed to passive smoking (at least one member of my family is a smoker).
	No.
height (in cm)	………..
Weight (in KG)	………..
Do you have any of the following diseases or conditions?	Diabetes
	Deviated nasal septum
	Allergic rhinitis
	Asthma
	Stuffy nose
	Nothing
The environment:	Pets
	Exposure to detergents
	Exposure to dust and plants.
	Exposure to gases and smoke
	Wet environment
	Nothing
